# Pulmonary artery aneurysm and hemoptysis as uncommon sequelae of COVID-19 infection: a case report from Syria

**DOI:** 10.1097/MS9.0000000000000822

**Published:** 2023-05-10

**Authors:** Hussein Hamdar, Ali Alakbar Nahle, Amjad Sikaria, Salem Algomaa Alhadid

**Affiliations:** a Faculty of Medicine, Damascus University; b Al Kindi Hospital, Damascus, Syrian Arab Republic

**Keywords:** case report, COVID-19, infection, pulmonary artery aneurysm

## Abstract

Pulmonary artery aneurysm (PAA) is a rare vascular disease, and it is known to be an extremely rare complication following infection with the coronavirus disease 2019 (COVID-19). Other etiologies of the disease are cancer, congenital heart anomalies, and vasculitis. While it is usually an asymptomatic disease, PAA can sometimes present with severe symptoms such as hemoptysis due to the rupture of the aneurysm.

**Case presentation:**

Here, The authors report a case of a 75-year-old female patient presented with hemoptysis. She had normal vasculature on a previous computed tomography scan when she was diagnosed with COVID-19. Four months after having COVID-19, and with a computed tomographic pulmonary angiography -assisted diagnosis, the patient was later diagnosed with PAA.

**Clinical discussion:**

PAAs have a wide nonspecific range of symptoms. The main diagnostic test is computed tomography angiography. Treatment is still controversial with no definite agreement on the management.

**Conclusion:**

COVID-19 infections can cause severe damage to blood vessels, especially in the context of other etiologies that can further damage the vasculature. This report demonstrates the importance of patient follow-up and monitoring post-COVID-19 infection in reducing further complications and mortality.

## Introduction

Highlights

- Pulmonary artery aneurysm is a rare vascular dysfunction. Pulmonary artery aneurysm is known to be an even rare complication post coronavirus disease 2019 (COVID-19) infection.
- A 75-year-old woman with a previous COVID-19 infection suffered from hemoptysis due to a ruptured aneurysm and underwent successful surgical lobectomy.
- COVID-19 complications and follow-up imaging are crucial, especially when coupled with vascular dysfunction-causing conditions like diabetes and hypertension.

Pulmonary artery aneurysms (PAAs) are an infrequent condition, accounting for ~1% of all intrathoracic aneurysms^1^. The development of PAA is an exceedingly rare complication following coronavirus disease 2019 (COVID-19) infection^2^. PAAs can arise from a variety of etiologies, including cancer, infection, congenital heart anomalies, or vasculitis^3^. While PAAs are often asymptomatic and discovered incidentally during imaging studies, they can also lead to serious health consequences such as pulmonary artery (PA) dissection, massive hemoptysis from rupture, and acute coronary syndrome resulting from compression of the coronary artery^3^. Rupture of a PAA can cause significant lung hemorrhage leading to asphyxia and aspiration, which are the primary causes of mortality in these patients. The reported mortality rate following rupture of a PAA varies between 50–100%^4,5^.

This work has been reported in line with the Surgical CAse REport (SCARE) 2020 criteria^6^.

## Case presentation

**Figure 1:**
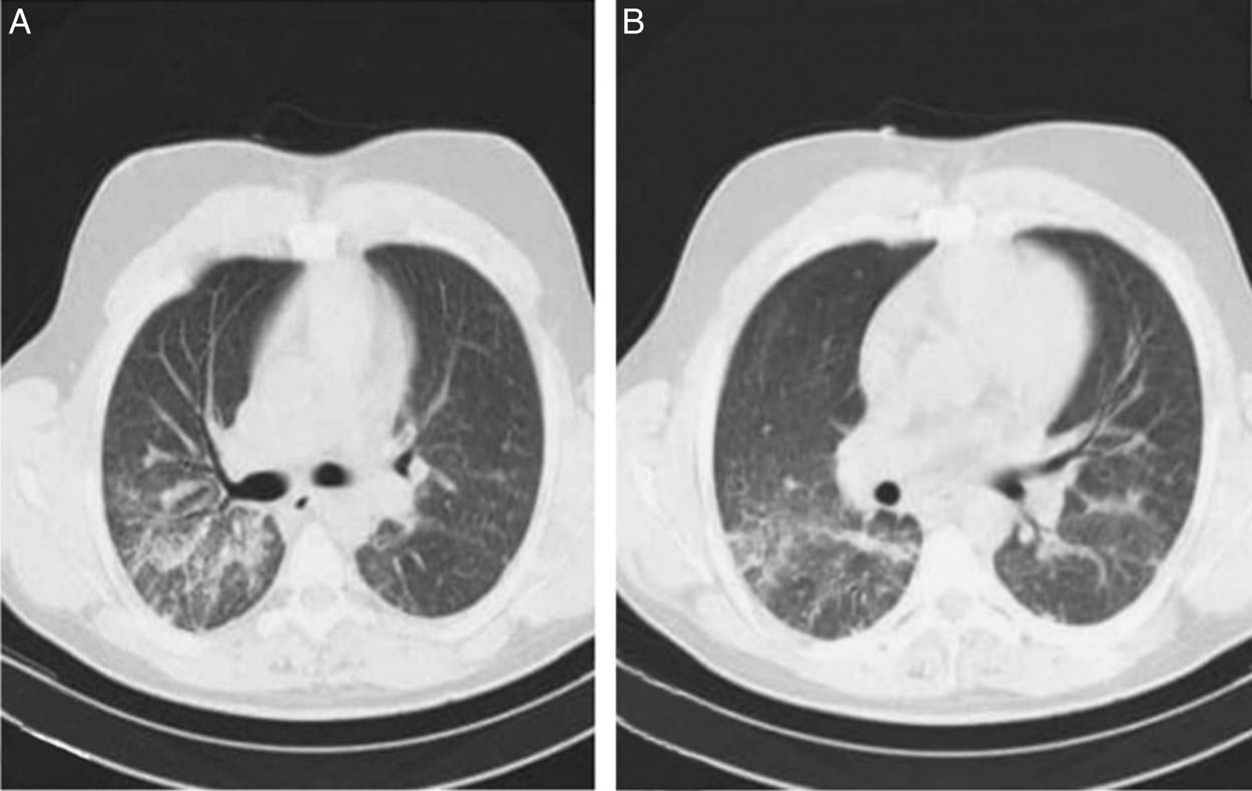
(A, B): Computed tomography scan showing diffuse pulmonary infiltrates most consistent with coronavirus disease 2019 and no presence for an aneurysm.

**Figure 2:**
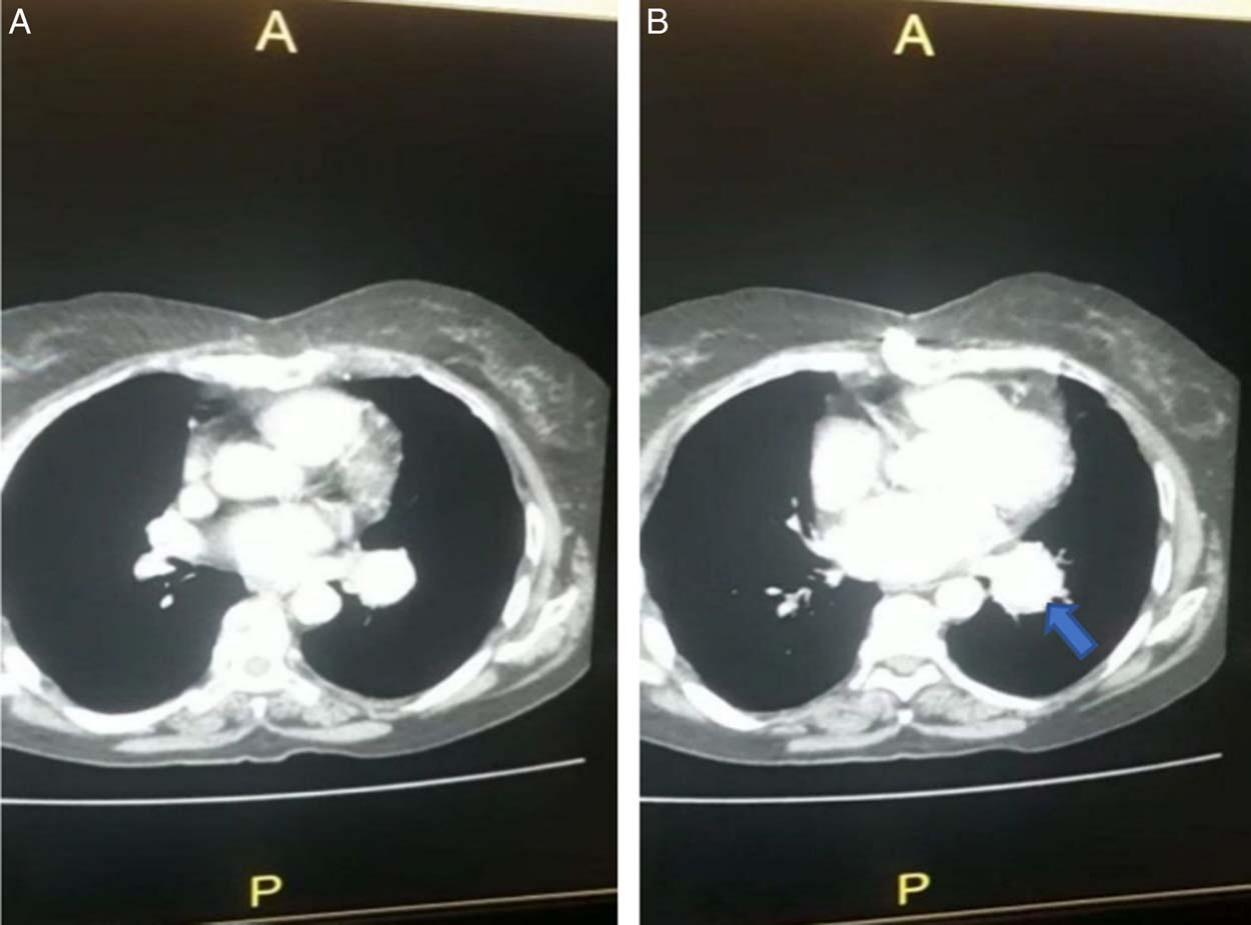
(A, B): Computed tomography pulmonary angiogram showing a lesion in the hilum of the left lung measuring 37×52 mm and multiple cavitary consolidations in direct contact with the segmental branch of the left pulmonary artery in the lower left lobe representing pulmonary artery aneurysm (arrow).

A 75-year-old female Eastern Mediterranean patient with a history of COVID-19 presented to our hospital after experiencing four episodes of hemoptysis with no other associated symptoms. There was no evidence of fever, dyspnea, chest pain, palpitations, or syncopal episodes. The patient had a medical history of smoking, diabetes mellitus, hypertension, and coronary artery bypass grafting surgery. The diagnosis of COVID-19 was confirmed in late November 2021 through a SARS-CoV-RNA transcription-mediated amplification test and chest computed tomography (CT) scan (Fig. 1 A, B). The CT scan showed diffuse pulmonary infiltrates consistent with COVID-19, and no aneurysm was detected at that time. The patient was treated with supplemental oxygen, anticoagulation, and steroids. Four months after the COVID-19 illness, she developed generalized fatigue, a productive cough, and four episodes of hemoptysis with ~50 ml of blood per episode. The patient was referred to our department for symptom management. During the physical examination of the chest, an inspection was made that the chest was symmetrical and there were no external deformities. Further examination revealed normal breath sounds upon auscultation and no masses were detected during palpation. A complete blood count showed a hemoglobin level of 8 g/dl, but liver and renal functions were normal. A computed tomographic pulmonary angiography was performed, which revealed a lesion in the hilum of the left lung measuring 37×52 mm extending to the lower lobe. The lesion showed clear and regular margins in direct contact with the segmental branch of the left PA in the lower left lobe, suggesting the presence of a PAA. The computed tomographic pulmonary angiography also showed multiple areas of consolidations in both lungs (Fig. 2 A, B). Sputum was negative for acid-fast bacilli, so a bronchoscopy was performed to complete the tuberculosis (TB) study and determine the origin of hemoptysis. Blood was present in the left lower lobe bronchus, and bronchoalveolar lavage was studied with a negative result for TB. Furthermore, an echocardiogram showed normal heart function with an ejection fraction of 55%. Subsequently, surgical intervention was decided upon.

**Figure 3:**
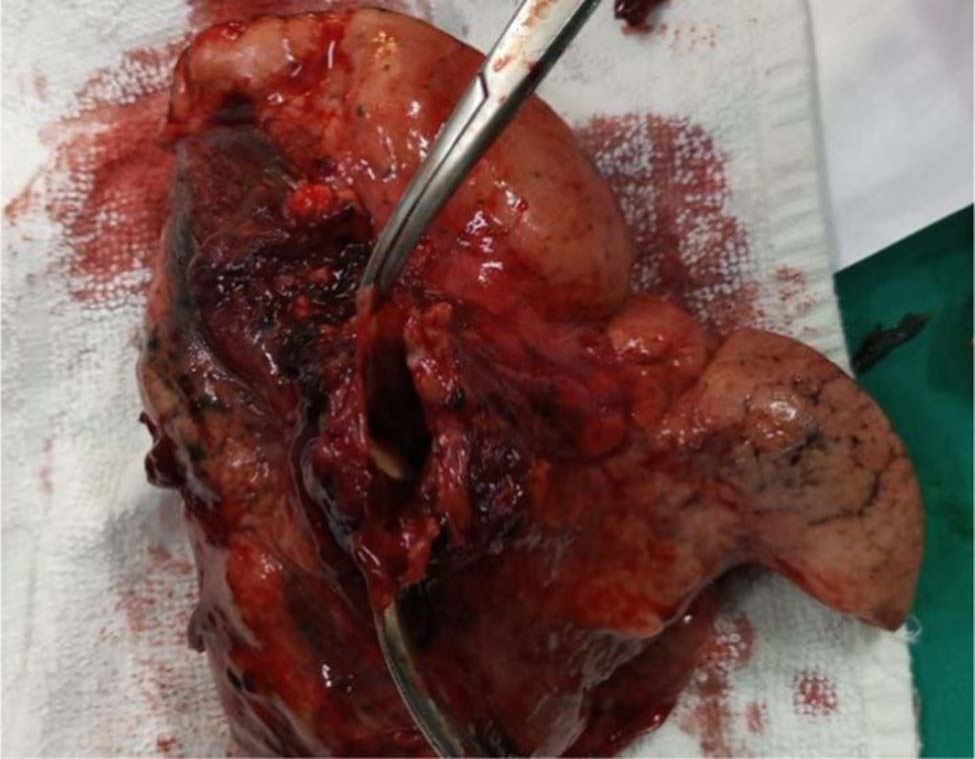
Resected left inferior lobe showing the lumen of the aneurysm.

The surgery was performed at a moderately developed public hospital by a thoracic surgeon with 7 years of experience. It was performed after correcting the low hemoglobin level by blood transfusion and reaching a level of hemoglobin 9.5 g/dl. The surgery was carried out through a left lateral thoracotomy in the fifth intercostal space under general anesthesia. The left inferior lobe, including the aneurysm, was resected (Fig. 3), after ligating the inferior pulmonary vein and the branches of the left PA. The bronchus of the inferior lobe was also resected, sutured, and a chest tube was inserted. The surgical procedure was executed without any complications or unfavorable outcomes during and post-operation.

Pathological examination revealed foci of a large aneurysm in the inferior lobe of the left lung measuring 5 cm, which was compatible with post-COVID-19 infection. Foci of granuloma with necrosis near the pleura were evident, suggesting the presence of TB, and no atypical cells were seen. The patient received antituberculous drugs (Isoniazid 300 mg, Rifampin 600 mg, pyrazinamide 2 g, and ethambutol 1.2 g daily) for 6 months.

**Figure 4:**
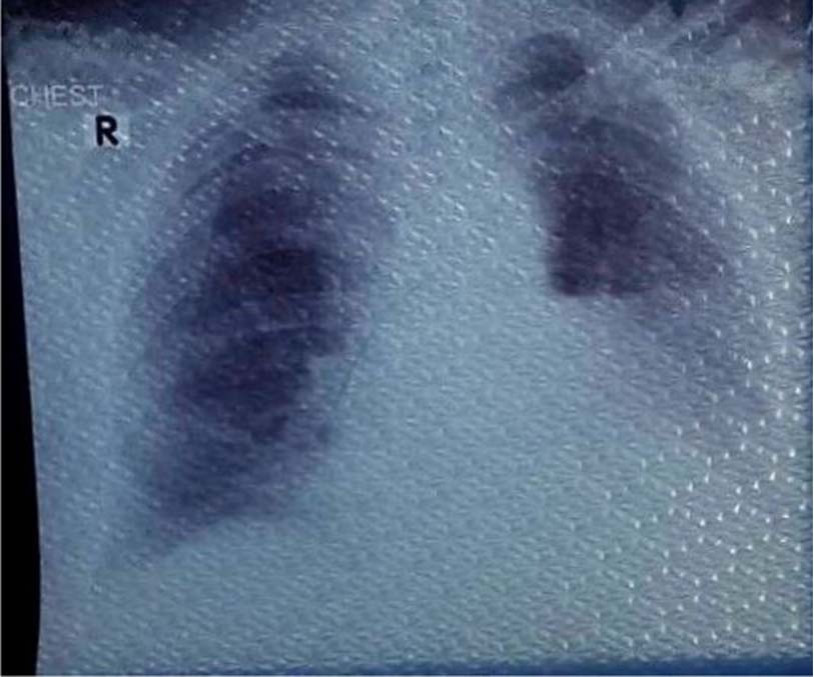
A follow-up chest radiography was performed one week postsurgery, which revealed the presence of a pleural air-fluid level on the left side.

**Figure 5:**
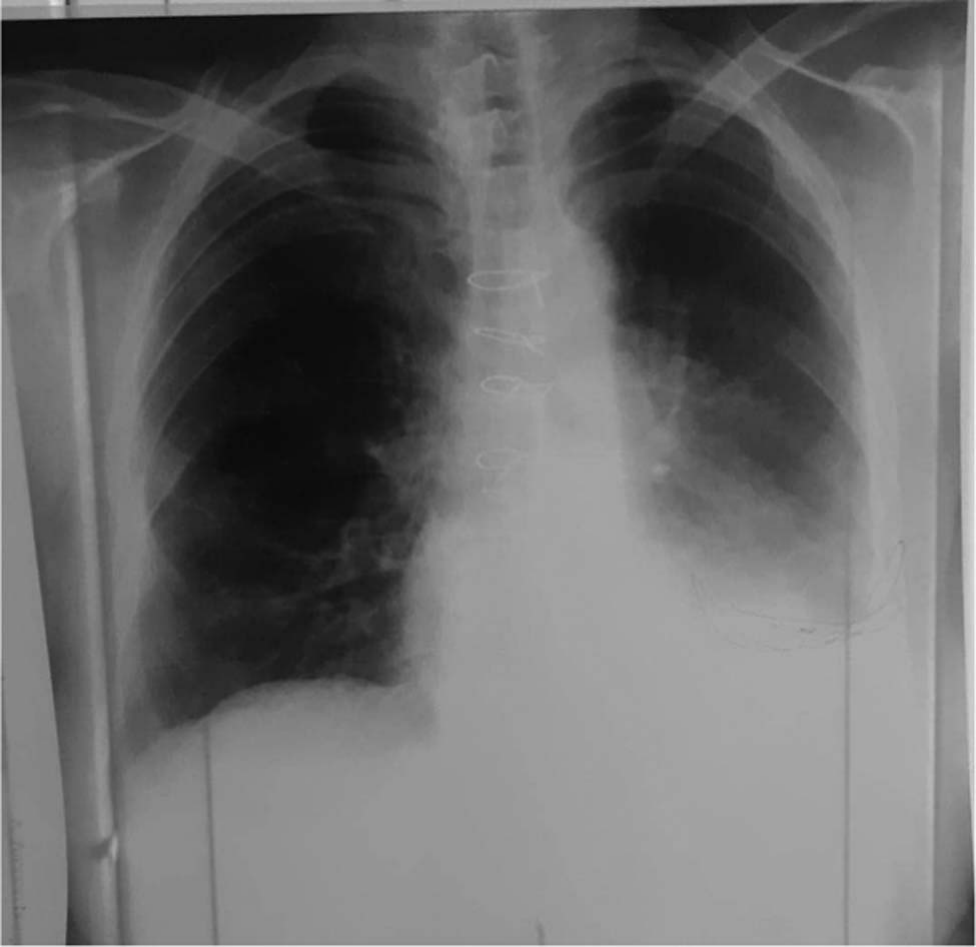
A follow-up chest radiography performed two weeks postsurgery showed normal findings.

Upon postoperative evaluation, the patient’s hemoptysis was noted to have ceased. Post-operative recovery was well-managed, with special attention given to pain management, mobilization, respiratory care, and nutritional support. The patient was closely monitored during the recovery period to ensure proper healing and minimize any potential complications. Overall, the patient responded well to the postoperative care, and the recovery was uneventful. Following removal of the chest tube, the patient was discharged in a stable condition. A week later, a follow-up chest radiography revealed the presence of a pleural air-fluid level on the left side (Fig. 4). The patient was subjected to close monitoring, and a week later, the pleural air-fluid level was observed to have spontaneously resolved, with normal findings noted on a subsequent chest radiography (Fig. 5). The patient was followed up for a period of 2 months, during which there was no recurrence of hemoptysis or any indications of postoperative complications. The patient was found to be in good overall health, and no significant pain or discomfort was reported.

## Discussion

PAA is an uncommon pathological condition^7^, wherein the PA undergoes abnormal dilation involving all three layers of its wall. Conversely, pseudoaneurysms lack all three layers and exhibit higher susceptibility to ruptures than PAA^8^. In the present case, the rupture of the PAA resulted in a severe clinical presentation.

Several factors have been identified as potential causes of this disease, including congenital abnormalities, infections, PA hypertension, and vasculitis^7,9,10^. The etiology of infection in this disease involves disruption of the vascular wall, which makes it more susceptible to dilation and aneurysm formation^11,12^. Previous research has established a correlation between vascular damage in the pulmonary arteries and concomitant infections of TB and COVID-19^9^. Another study has found that the COVID-19 pandemic has resulted in delayed diagnosis and management of TB, contributing to an increase in the incidence of vascular damage in the pulmonary arteries^13^. In the present case, the patient had COVID-19 infection and subsequently developed an aneurysm 4 months later despite initially having normal pulmonary vasculature. Earlier research suggests that the infection caused by COVID-19 has the potential to cause vascular dysfunction^14^. Additionally, the pathology report following the surgery revealed areas of granuloma with necrosis, indicating the potential presence of TB, despite the negative results from preoperative sputum tests and bronchoalveolar lavage. This gives rise to the hypothesis that our patient may have had a concurrent infection with both TB and COVID-19, which could have resulted in more severe vascular damage. The use of steroids may lead to a state of generalized immunosuppression, which can elevate the likelihood of latent TB infection reactivation^15^. In our case, the patient was administered steroids medication when infected with COVID-19, which has the potential to reactivate TB and exacerbate damage to the vessels.

PAAs exhibit a broad and nonspecific spectrum of symptoms, which may even be asymptomatic. The non-specific symptoms occur as a result of the compression of neighboring structures^16^. These symptoms consist of dyspnea, chest pain, hoarseness, palpitation, and syncopal episodes^10,17–22^. Additionally, sudden cardiac death may also occur as a manifestation of the disease^23^. Hemoptysis, caused by aneurysm rupture as shown in our case, is another symptom that can present in patients with PAAs^23^. In elderly patients presenting with hemoptysis, various underlying causes are considered, such as bronchiectasis, hydatid cyst, TB, and pulmonary edema. It has been noted that TB and pulmonary edema are more common in patients over 65 years old^24^. However, in the post-COVID-19 era, it is important to also consider rupture of PAA as a differential diagnosis in this patient population as our case demonstrates.

PAA is often detected incidentally via chest radiography or transesophageal echocardiography, as it typically presents without symptoms, CT angiography is then used to confirm diagnosis^25,26^. The management of PAA remains controversial, with no definitive consensus on treatment^27^. Conservative options, such as antibiotics, immunosuppressants, and glucocorticoids, are typically preferred for PAA caused by secondary medical conditions^25^. However, nonconservative interventions, such as surgery and interventional procedures, are generally considered in cases of rupture as was the case in our article or in symptomatic patients^3,20^. Due to the high mortality and complication risks associated with surgery, interventional treatments are often preferred over surgical options^27^. In this particular case, surgery was successfully performed due to the unavailability of interventional treatments at the hospital.

Khurram *et al*.^14^ were the first to report a case of PAP on a COVID-19 background. In our knowledge herein, we have reported the first case of PAA occurring after COVID-19 infection with a 4-month interval and severe complications. This provides further evidence of the serious vascular dysfunction that can result from COVID-19 infection. As hypertension and diabetes are well-known contributors to vascular dysfunction^28^, these comorbidities may have exacerbated the severity of the COVID-19 infection on our patient’s blood vessels. Therefore, we strongly recommend that patients with COVID-19 infection be closely monitored for any vascular damage, especially those with pre-existing hypertension, diabetes, and concurrent TB infection in order to prevent potential complications.

## Conclusion

In this report, we have presented a case of a ruptured PAA following a COVID-19 infection that was successfully managed with a surgical lobectomy. Since a prior infection of COVID-19 increases the susceptibility of vascular damage and dysfunction, it is recommended to monitor patients who have been infected with COVID-19 and have diseases associated with vascular dysfunction in order to prevent further complications and mortality.

## Ethics approval and consent to participate

Not applicable.

## Consent for publication

Written informed consent was obtained from the patient for publication of this case report and accompanying images. A copy of the written consent is available for review by the Editor-in-Chief of this journal on request.

## Sources of funding

Not applicable.

## Authors’ contributions

H.H. is the first author contributed to drafting, reviewing and editing, corresponding, and bibliography. A.A.N. is a co-first author, contributed to drafting, reviewing and editing. A.S. contributed to reviewing, and editing. S.A.A. contributed to reviewing, editing, and supervising. All authors read and approved the final manuscript.

## Conflicts of interest disclosure

The authors declare that they have no competing interests.

## Provenance and peer review

Not commissioned, externally peer reviewed.

## Guarantor

Dr. Salem Algomaa Alhadid.

## Availability of data and materials

Not applicable.
